# Augmented reality-assisted systematic mapping of anterolateral thigh perforators

**DOI:** 10.1186/s12891-022-06013-1

**Published:** 2022-12-02

**Authors:** Yifu Tang, Qiang Guo, Xiaoning Li, Yuzhao Huang, Wei Kuang, Ling Luo

**Affiliations:** Department of Orthopaedics, The Third Xiangya Hospital, Central South University, No. 138, Tongzipo Road Changsha, Hunan, China

**Keywords:** Augmented reality, Vascularized flaps, Soft tissue reconstruction, Microsurgery, Computed tomography angiography

## Abstract

**Purpose:**

In soft tissue reconstructive surgery, perforator localization and flap harvesting have always been critical challenges, but augmented reality (AR) has become a dominant technology to help map perforators.

**Methods:**

The lateral circumflex femoral artery (LCFA) and its perforators were reconstructed by CTA in consecutive patients (*N* = 14). Then, the anterolateral thigh perforators and the points from which the perforators emerged from the deep fascia were marked and projected onto the skin surface. As the virtual images were projected onto patients according to bony markers, the courses of the LCFA and its perforators were depicted on the skin surface for intraoperative guidance. Finally, the locations of the emergence points were verified by intraoperative findings and compared to those determined by handheld Doppler ultrasound.

**Results:**

The sources, locations, and numbers of perforators were determined by CTA. The perforators and their emergence points were accurately mapped on the skin surface by a portable projector to harvest the anterolateral thigh perforator flap. During the operation, the accuracy of the CTA & AR method was 90.2% (37/41), and the sensitivity reached 97.4% (37/38), which were much higher than the corresponding values of Doppler ultrasound. Additionally, the differences between the AR-marked points and the intraoperative findings were much smaller than those seen with Doppler ultrasound (*P* < 0.001). Consequently, all of the flaps were well designed and survived, and only one complication occurred.

**Conclusion:**

Augmented reality, namely, CTA combined with projection in this study, plays a vital and reliable role in locating the perforator emergence points and guiding the procedure to harvest flaps and has fewer potential risks.

## Introduction

In orthopedics, debridement and soft tissue reconstruction are of equal importance in managing open fractures, aiming to remove the contaminated tissues and facilitate fracture healing. The most common methods for closing open wounds include primary closure, skin grafts, local flaps, and free flaps [1]. Among them, flap transplantation has incomparable advantages in treating complex open fractures with large soft tissue defects, heavy contamination and exposure of vital tissues. To our knowledge, the anterolateral thigh perforator (ALTP) flap has been one of the most versatile and effective flaps to treat complex soft tissue defects in orthopedics and has the following benefits: long and constant vascular pedicles, high maneuverability, large flap area, low impact to the donor area, and the ability to encompass fascial, muscular, and neural tissues to handle various soft tissue defects [2]. However, complicated issues remain, involving large variability of the vessels, long operating time, and mastery of the required surgical skills. Consequently, even excellent microsurgeons need to spend considerable time and effort harvesting free flaps. Inevitably, perforator injury and vascular crisis are difficult to avoid when separating tiny perforators. Moreover, uncertainty in perforator distribution can lead to discrepancies in the blood flow of the harvested flaps, causing partial flap necrosis and even surgical failure [2]. Therefore, improving the surgical efficiency and flap survival rate has become an urgent problem.

To address these problems, novel imaging techniques have been explored to locate perforators and harvest various flaps. Handheld Doppler ultrasonography was first applied to display real-time perforator distribution and has the advantages of being convenient and economical [3]. This method requires little expertise and is simple to use, although smaller perforators are easily overlooked and often affected by deep arterial pulsations. Therefore, handheld Doppler ultrasonography should be used with caution for flap design, preferably as a supplement or reference for the rough positioning of the perforators. With the development of imaging techniques, magnetic resonance angiography (MRA) has shown unique advantages for locating tiny perforators, but it does not meet the requirements of efficiency and applicability [4]. Therefore, it is not suitable for promotion.

As the current strategies are insufficient to obtain the necessary information related to perforators, there is an urgent clinical need for a method to locate the sources, courses, and exact numbers of ALTPs with high accuracy and precision. Generally, augmented reality (AR) has been used in an increasingly wide range of applications to combine advanced imaging and projection techniques [5–7]. Among the available techniques, computed tomographic angiography (CTA) is considered the gold standard for detecting ALTPs [8, 9]. Shen used CTA in combination with an equivalently sized template to preoperatively reproduce the ALTP to improve the efficiency of intraoperative flap harvest, and the method was more accurate and comprehensive than the commonly used portable ultrasound [10]. In addition, Hummelink performed cosmetic breast reconstruction after radical tumor surgery by using CTA combined with projection techniques by reproducing images of the deep inferior epigastric perforator (DIEP) preoperatively [11].

However, the CTA-related projection technology in previous studies is still affected by a few deviations, and many details should be modified before adaptation for harvesting flaps. First, the CTA scan parameters should be optimized to achieve higher accuracy, allowing the tiny caliber of perforators to be detected. Second, small vessels need to be dilated safely, and the delay time should be extended during CTA examination to obtain more perforator details. Moreover, the errors arising from projection can be significantly reduced by locating consistent bony markers, such as the anterior superior iliac spine (ASIS) and patella. Most importantly, 80% of ALTPs are musculocutaneous, and they are frequently damaged when dissected from muscles and deep fascia during the process of flap harvest. Thus, the key point of this paper is to precisely locate these vulnerable “perforator emergence points”. Given that fat and muscle tissue can be differentiated on CT [12], the interface between muscle and fat could be considered the deep fascia layer, and the points representing perforators emerging from the deep fascia could be easily marked. Consequently, this new method relying on the combined CTA & AR technique helps us locate the emergence point of the perforators more accurately and sensitively, thus allowing for precise flap design and improved flap survival.

## Methods

### General information

Approval from the ethical committee of The Third Xiangya Hospital was obtained, and informed consent was acquired from the patients. Between January 2020 and July 2021, we studied a prospective cohort of 14 consecutive patients who underwent preoperative CTA mapping of their ALTPs; subsequently, the location of the emergence points were accurately determined using the projection technique intraoperatively. We excluded patients who had a history of allergic reactions to CT contrast agents, renal failure, or severe cardiovascular disease, as these could be high-risk factors for the CTA procedure. Before the operation, handheld Doppler ultrasound was performed to locate the ALTP distribution as a control. Fourteen patients (13 male and 1 female) ranging in age from 29 to 69 years (mean age: 50.8 years) were involved.

### CTA mapping of the emergence points of the ALTPs

**Fig. 1:**
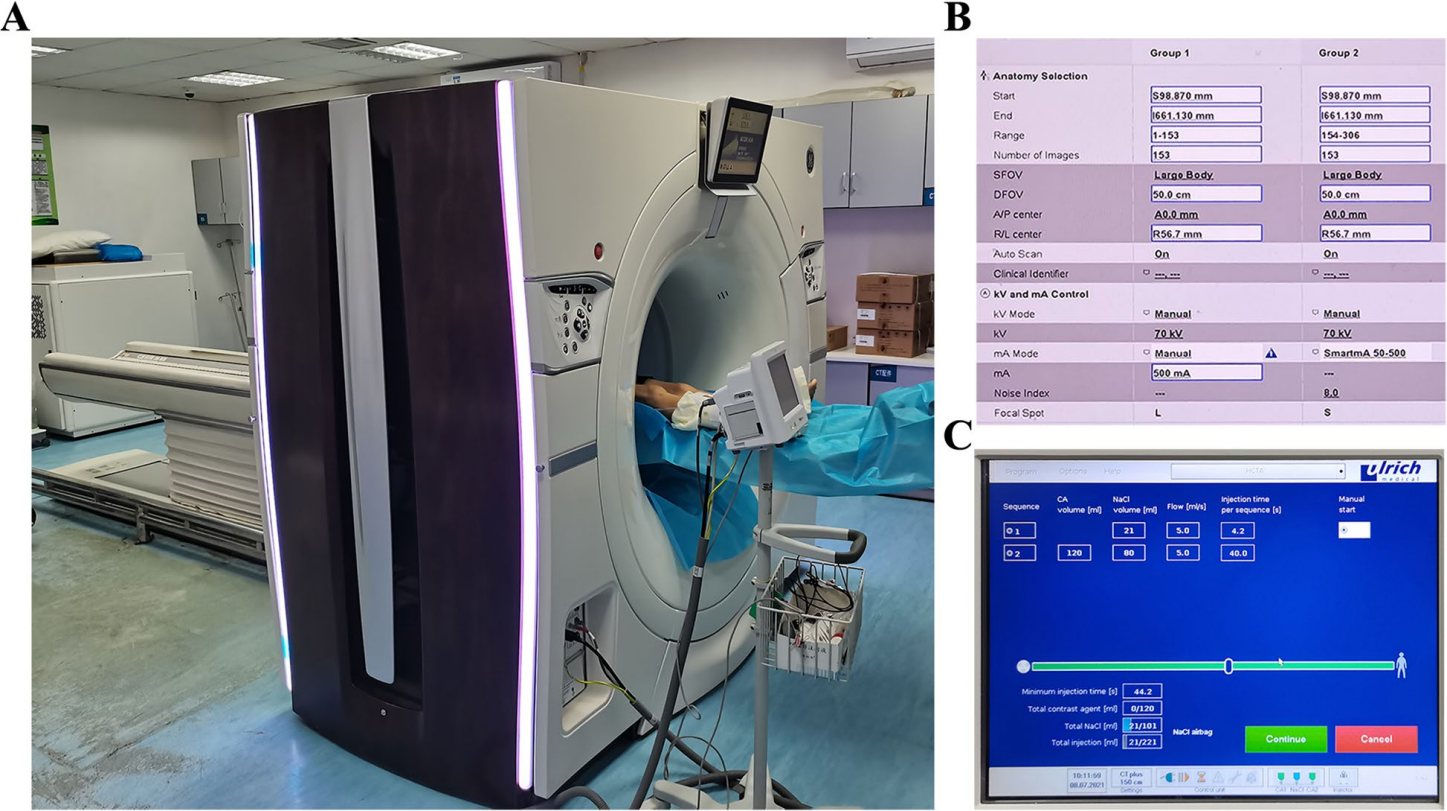
CTA examination and its parameters. **A** Patients were scanned by GE medical systems revolution CT. **B** The CTA parameters were set as lower kilovolt (70 kV) and higher mA value (500 mA). **C** The injection rate and total volume of iodinated contrast medium

**Fig. 2:**
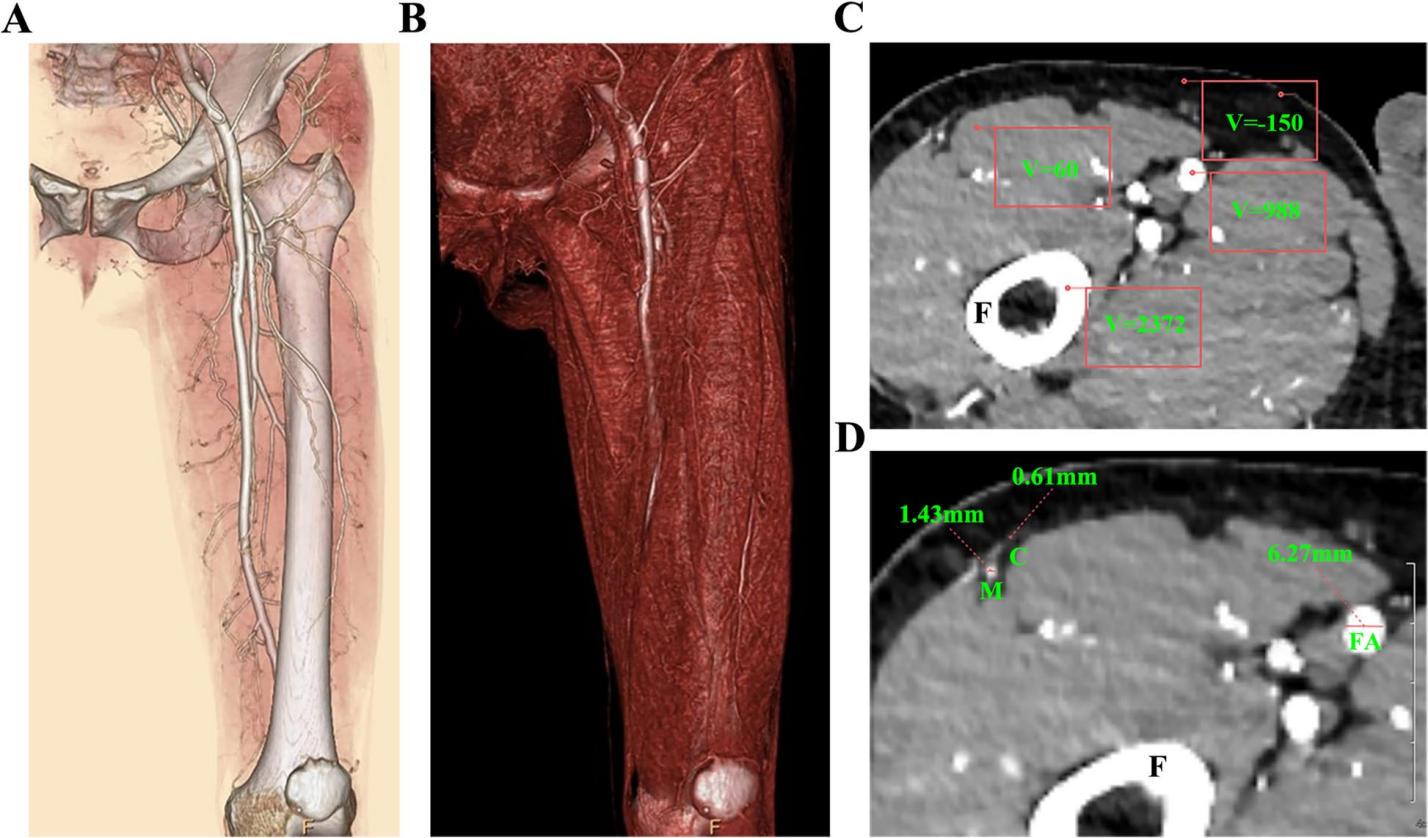
Three-dimensional reconstructed CTA images of the thigh. **A** Region of interest ranging from ASIS to patella was selected to reveal the whole view of thigh and ALTP. **B** Volume rendering of the muscles and artery, to display the perforators emerging from the deep fascia. **C** Different tissues possess different Hounsfield unit (HU) values in CT scan (Bone: 2000-2500HU, Artery: 800-1200HU, Muscle: 50-80HU, Fat: under -140HU). **D** The diameters of femoral artery, descending branch, perforators, and their cutaneous branches

**Fig. 3:**
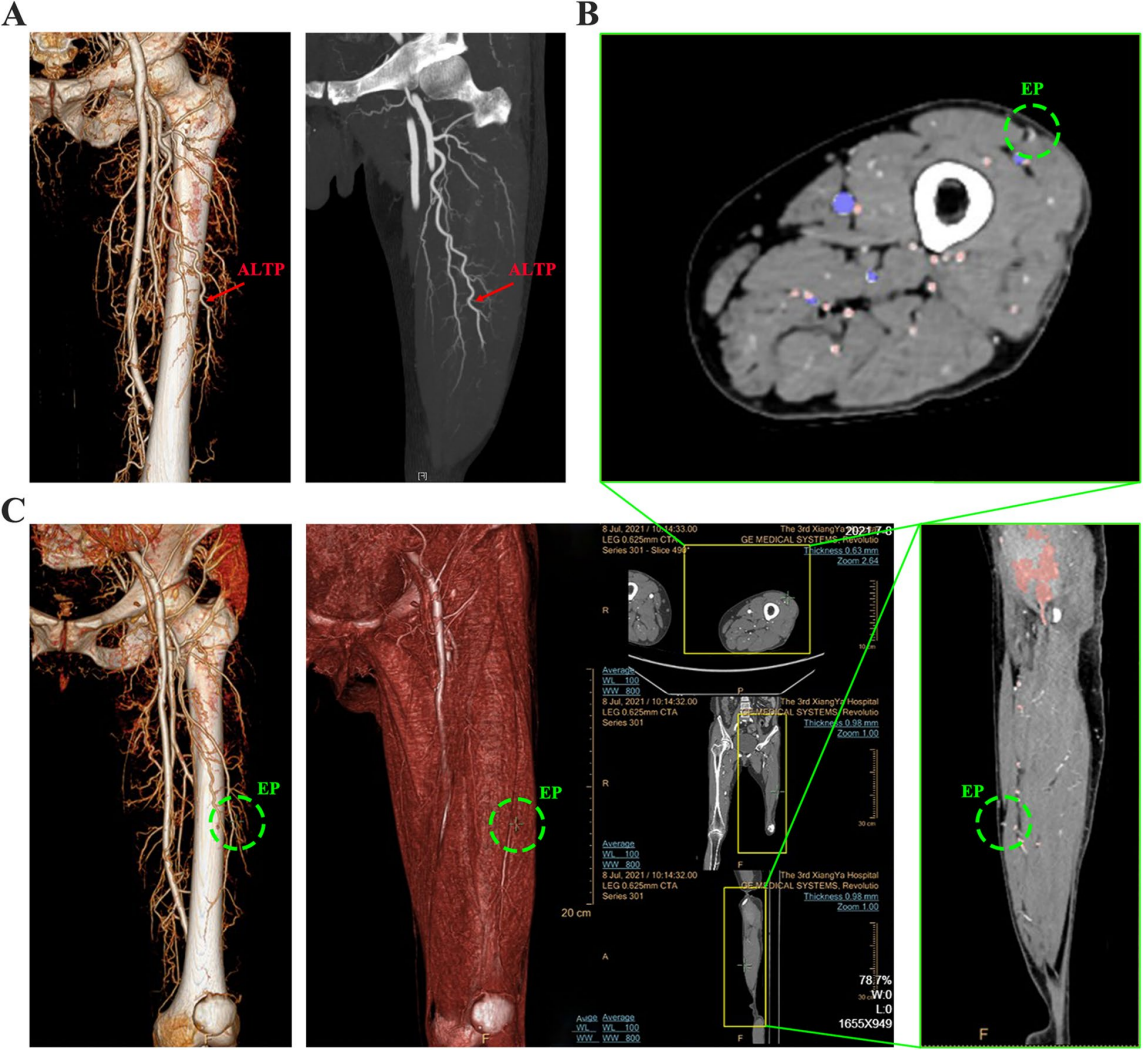
Detailed process for obtaining ALTP emerging points. **A** The whole course, including descending branch of femoral artery and ALTP, were reconstructed by software. **B** The emerging points were firstly identified and marked by checking every transverse slice. **C** At this time, the corresponding points in the coronal, sagittal plane and 3D images were automatically marked by the software. The emerging points of perforators came out from the muscle surface were also displayed in the volume rendering 3D image. ALTP: anterolateral thigh perforator, EP: emerging point. Identity information has been removed to ensure patient anonymity

The GE medical systems revolution CT (GE Healthcare, Waukesha, WI, USA) was utilized in our study (Fig. 1A), and the parameters are as follows (Fig. 1B): 70 kVp, 500 mA, gantry rotation time of 1.0 s, and matrix 512 × 512. The patients were given 0.5 mg of sublingual nitroglycerin (Xinyi Corporation, Shandong, China) before the CT scan to appropriately dilate the perforators. During the examination, the patients were placed in the supine position, while iodinated contrast medium (100–120 ml) was injected at a rate of 5.0 ml/s into the antecubital vein (Fig. 1C). A region of interest ranging from the ASIS to the patella was selected to reveal the whole view of the ALTP, and the slice width was set as 0.625 mm to obtain the acquired volumetric data. Then, the images were transferred to a Philips Intelli Space Portal (Philips, N.V. Netherlands) automatically, and various required three-dimensional (3-D) images were generated to display the descending branch and its perforators (Fig. 2A, B). Moreover, different Hounsfield unit (HU) values of various tissues and calibres of vessels were detected (Fig. 2C, D). Principally based on the transverse images, the emergence points of the perforators from the muscle or intermuscular septum were marked and could be synchronously displayed in the coronal, sagittal and 3-D CTA images. Moreover, to reveal the emergence point of the ALTP more directly, image processing was performed to preserve the quadriceps femoris, and the subcutaneous fat tissue was removed (Fig. 3). Finally, these marked images were imported to the portable projector for the preoperative depiction of the ALTPs.

### Handheld doppler ultrasound

The patient was kept in the supine position, and the bony markers (ipsilateral ASIS and patella) were palpated. The course of the femoral artery was depicted, and a line connecting the ASIS and the lateral border of the patella was drawn. Then, to detect the perforator distribution of the lateral circumflex femoral artery (LCFA), the midpoint of the ASIS-patellar line was also marked. The coupling agent was evenly applied to the skin surface of the thigh, and a portable ultrasound (ES-100V3, Hadeco, Inc., Kawasaki, Japan) probe was placed vertically, in slight contact with the skin surface, to detect the pulsation of the possible perforators. Finally, these specific points were marked to assist with intraoperative flap harvest.

### Augmented reality projection and depiction

**Fig. 4:**
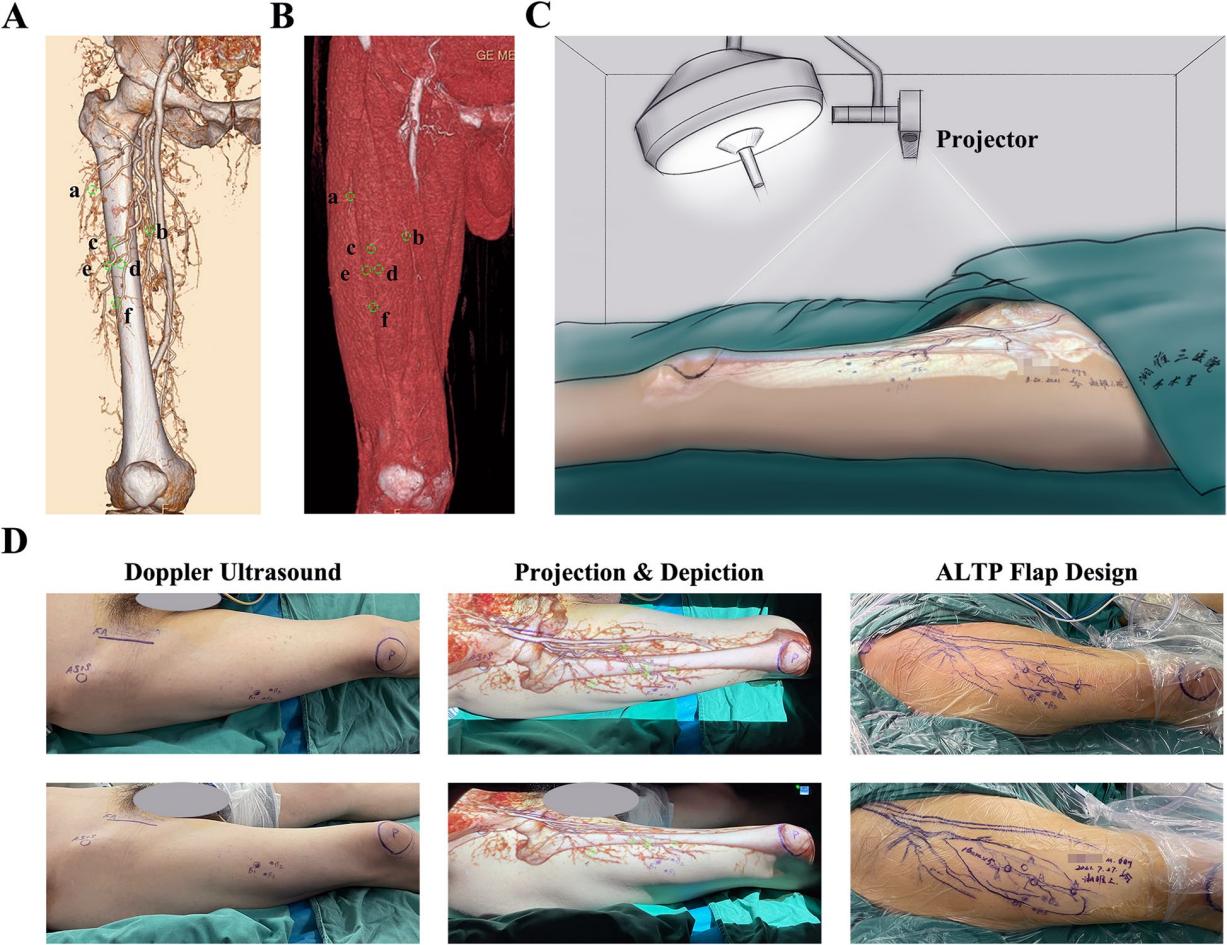
Augmented reality (AR) for ALTP flap design. **A** The points a-f were marked on the 3D CTA images. **B** The points a-f were marked on the muscle surface. **C** The marked images were imported in the portable projector, and then mapped onto the thigh surface before operation. **D** Doppler ultrasound and AR were used to depict the perforators and emerging points, to facilitate the ALTP flap harvest

The prepared CTA images (Fig. 4A, B) were imported into the portable projector (XE11F, XGIMI Co., Chengdu, China), and the bony markers (the outline of ASIS and patella) in the images were projected coincident with the premarked bone contours (Fig. 4C). During this process, the position of the projector could be adjusted manually to align the ASIS and patella in the virtual map with the markers in the real surgical field. The projector could automatically adjust the focal length. At this time, the course of the LCFA and its descending branches was depicted on the skin surface, while the emergence points indicating the origin of the perforators from the deep fascia were also marked on the anterolateral thigh. Thus, the intraoperative ALTP flap area was designed to ensure sufficient blood supply (Fig. 4D).

### Preoperative design and intraoperative exploration

**Fig. 5:**
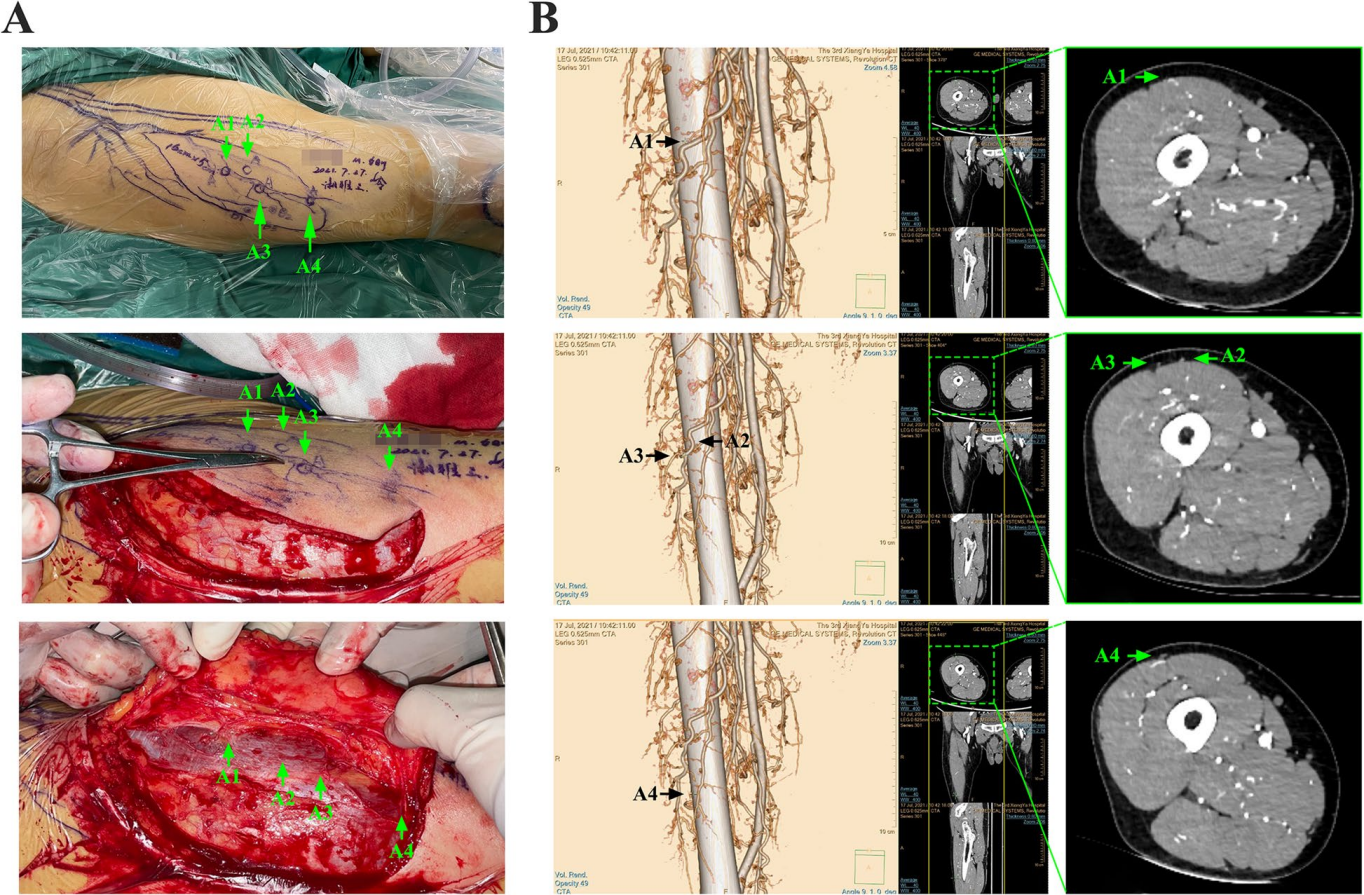
The ALTP flap was obtained intraoperatively by mapping the emerging points. **A** The four emerging points A1-A4 were depicted pre-operation, and verified intra-operation. **B** These emerging points were firstly found in transverse slice (on the rightmost), and then they were marked in three plane and 3D CTA images automatically. Identity information has been removed to ensure patient anonymity

Before the operation, the descending branch of the LCFA and its perforators, as well as the emergence points, were marked and depicted on the lateral thigh. Following disinfection and draping, the soft tissue defect was first measured. Then, the potentially contaminated area of each recipient was immediately wrapped with a sterile cloth to avoid cross-infection. The flap was designed with the consideration of the localized emergence points of the perforators to guarantee sufficient blood supply to the ALTP flap. Afterwards, the skin and subcutaneous tissue were incised along the internal or lateral edge of the predesigned flap until reaching the deep fascia. Then, the subcutaneous tissue was carefully separated under a microscope, identifying the perforators based on the premarked emergence points and preserving the soft tissue sleeve of all perforators (Fig. 5). Moreover, every distance between the premarked points and intraoperative findings was measured. Briefly, at the point where the perforators arising from the deep fascia were discovered during the operation, a vascular clamp was used to make a small protrusion on the skin surface. Hence, the distance between the protrusion point and the pre-marked point (whether by the Doppler or CTA&AR method) was measured to determine the accuracy of the preoperative localization. After all the perforators were separated, the descending branch of the LCFA was revealed along with the intermuscular space between the rectus femoris and lateralis muscle. The perforators of the descending branch, including the musculocutaneous and septocutaneous perforators, were separated posteriorly, while the redundant perforators were ligated to harvest the ALTP flap.

### Statistical analysis

All data are shown as the means and standard deviations (M ± SD) and were analyzed by SPSS 25.0 (Chicago, IL, USA). An unpaired two-tailed Student's t test was used to determine statistical significance between the two groups. One-way ANOVA with Tukey’s post hoc test was used for multiple-group comparisons. Enumeration data were analyzed using the chi-square test. The significance level was set at *P* < 0.05.

## Results

### General information

As shown in Table 1, of the 14 patients, 13 (92.9%) were male, and 1 (7.1%) was female, with a mean age of 50.8 years. Among them, 13 patients had severe open injuries of the lower extremities (Gustilo IIIB, IIIC), and only 1 had scarring of the hand. Half of them had a Mangled Extremity Severity Score (MESS) less than 7, and the other half of the patients had scores greater than 7, meeting the criteria for amputation. However, the patients opted for an ALTP flap to repair the large soft tissue defect to preserve the damaged limb. Postoperatively, patient 12 had a mixed infection, in which the causative bacteria were Enterobacter cloacae and Proteus vulgaris, because of the heavily contaminated wounds. Fortunately, the wound was largely healed after dressing changes were performed for approximately 1 month.

**Table 1:** General information and operative details

Patient	Age	Sex	Limbs	Gustilo type	MESS	Flap size	Donor site	Complication
1	69	M	Calf, L	III B	6	18.0*7.0 cm	R	None
2	48	M	Foot, R	III C	10	14.0*6.0 cm	L	None
3	60	M	Foot, R	III C	9	14.5*8.0 cm	L	None
4	29	M	Foot, L	III B	8	12.0*5.5 cm	R	None
5	52	M	Foot, L	III B	6	13.5*9.5 cm	R	None
6	53	M	Foot, R	III C	5	11.5*8.0 cm	L	None
7	42	M	Calf, L	III B	5	20.0*11.0 cm	R	None
8	32	M	Calf, L	III B	9	17.5*13.0 cm	R	None
9	52	M	Calf, R	III B	6	15.5*9.0 cm, 22.0*12.0 cm	L	None
10	50	M	Foot, L	III C	11	14.0*5.5 cm	R	None
11	51	M	Hand, R	Scar	-	8.0*1.8 cm, 8.0*1.7 cm	L	None
12	56	F	Foot, R	III C	10	18.0*5.5 cm	L	Mixed Infection
13	60	M	Foot, L	III C	10	16.0*5.0 cm	L	None
14	57	M	Calf, R	III B	6	8.0*6.0 cm	R	None

### Number of emergence points acquired from imaging techniques and intraoperative findings

After the CTA examination and volume rendering, possible perforators emanating from the descending branch of the LCFA were meticulously identified on every transverse section, and then the emergence points were marked. A total of 55 and 38 emerging points were identified by CTA and Doppler ultrasound in 14 patients, respectively. Among them, CTA and ultrasound found 41 and 34 emergence points in the predesigned flap area, respectively, while 38 emergence points were found intraoperatively (Table 2). In the 14 patients, most of them had 2 (*N* = 6) or 3 (*N* = 7) ultrasound-located points, while the number of CTA-detected points was predominantly 3 (*N* = 5) and 4 (*N* = 5). The differences between the intraoperative findings, and ultrasound findings were greater but not statistically significant (*P* = 0.22), and the difference between the CTA and intraoperative findings were also not statistically significant (*P* = 0.852) (Table 3).

**Table 2:** Number of emerging points, identified by preoperative imaging techniques

Patient	CTA		Doppler ultrasound		Intraoperative findings
	Total number of emerging points	Points in pre-designed flap area	Total No. of emerging points	Points in pre-designed flap area	
1	5	4	3	3	4
2	6	4	3	3	3
3	2	2	3	2	2
4	4	4	3	3	3
5	4	3	3	3	3
6	3	3	2	1	2
7	4	3	4	3	4
8	5	4	2	2	3
9	3	3	2	2	3
10	4	3	2	2	3
11	3	2	2	2	2
12	4	1	3	3	1
13	6	4	3	3	4
14	2	1	3	2	1
Total	55	41	38	34	38

**Table 3:** Number of the emerging points located by preoperative CTA & AR projection or Doppler ultrasound, compared with intraoperative findings

No. of emerging points	Doppler Ultrasound	CTA & AR projection	Intraoperative Findings
1	1	2	2
2	6	2	3
3	7	5	6
4	0	5	3
Versus intraoperative findings	*P* = 0.22	*P* = 0.852	

### ALTP emergence points information

Verified by intraoperative findings, the emergence points depicted by handheld Doppler ultrasound and CTA were estimated by statistical methods (Table 4). In brief, although a total of 38, 55 emergence points were explored by ultrasound and CTA, some emergence points were not explored intraoperatively because they were beyond the predesigned flap area. Respectively, Doppler ultrasound and CTA found 34 and 41 emergence points that could be intraoperatively identified. Of the 34 points localized by ultrasound, 6 were not included because they were too far away from the intraoperative findings (> 3 cm), whereas the other 28 points were identified as valid emergence points of the perforators. Ten of the 38 emerging points found intraoperatively were not detected by Doppler ultrasound. Thus, the accuracy of handheld ultrasound was 82.4% (28/34), and the sensitivity was 73.7% (28/38). In contrast, 4 of the 41 points localized by CTA were not confirmed intraoperatively, while the other 37 points were identified as valid emergence points of the perforators. Moreover, among the 38 points that were found intraoperatively, only one point was not found preoperatively by CTA. Therefore, the accuracy of the CTA & AR projection method was 90.2% (37/41), and the sensitivity reached 97.4% (37/38).

**Table 4:** Estimation of the ALTPs emerging points depicted by handheld Doppler ultrasound and CTA & AR method, verified by intraoperative finding

Emerging points information	Imaging technique	
	Doppler ultrasound	CTA & AR
Preoperative location overall	38	55
Not in the pre-designed flap area	4	14
Preoperative location in pre-designed flap area	34	41
Intraoperative confirmation^a^	28	37
No intraoperative confirmation	6	4
Preoperative missing^b^	10	1
Accuracy	82.4% (28/34)	90.2% (37/41)
Sensitivity	73.7% (28/38)	97.4% (37/38)

^a^Only if the distance is less than 3 cm, it is considered as “Intraoperative confirmation”. (The distance between preoperative marked points and intraoperative actual emerging points)

^b^Emerging points were not detected by preoperative imaging technique but were found during the operation

### Discrepancies in positioning

The distances between the preoperatively marked points and actual emergence points found intraoperatively were considered another important indicator for the accuracy assessment, and these data are listed in Table 5. The 28 points marked by handheld ultrasound were 1.97 ± 0.50 cm from the intraoperatively detected points, while the locations marked by CTA were much more accurate (*P* < 0.001), with a difference in distance of 0.53 ± 0.27 cm. Moreover, the distance variances were significantly smaller in the CTA group than in the ultrasound group; an F test to compare variances revealed a value of 3.359 (*P* = 0.0372). Thus, the emergence points obtained by the CTA & AR projection technique were more accurate than those obtained by Doppler ultrasound.

**Table 5:** Emerging points discrepancies between preoperative marked points and intraoperative findings

	**Distance (cm)**				
	M ± SD	N	95% CI (%)	*P* (t test)	*P* (F test to compare variances)
Doppler ultrasound	1.97 ± 0.50	28	1.68–2.25	< 0.001	0.037
Augmented reality	0.53 ± 0.27	37	0.37–0.68		

### Flap survival and follow-up

**Fig. 6:**
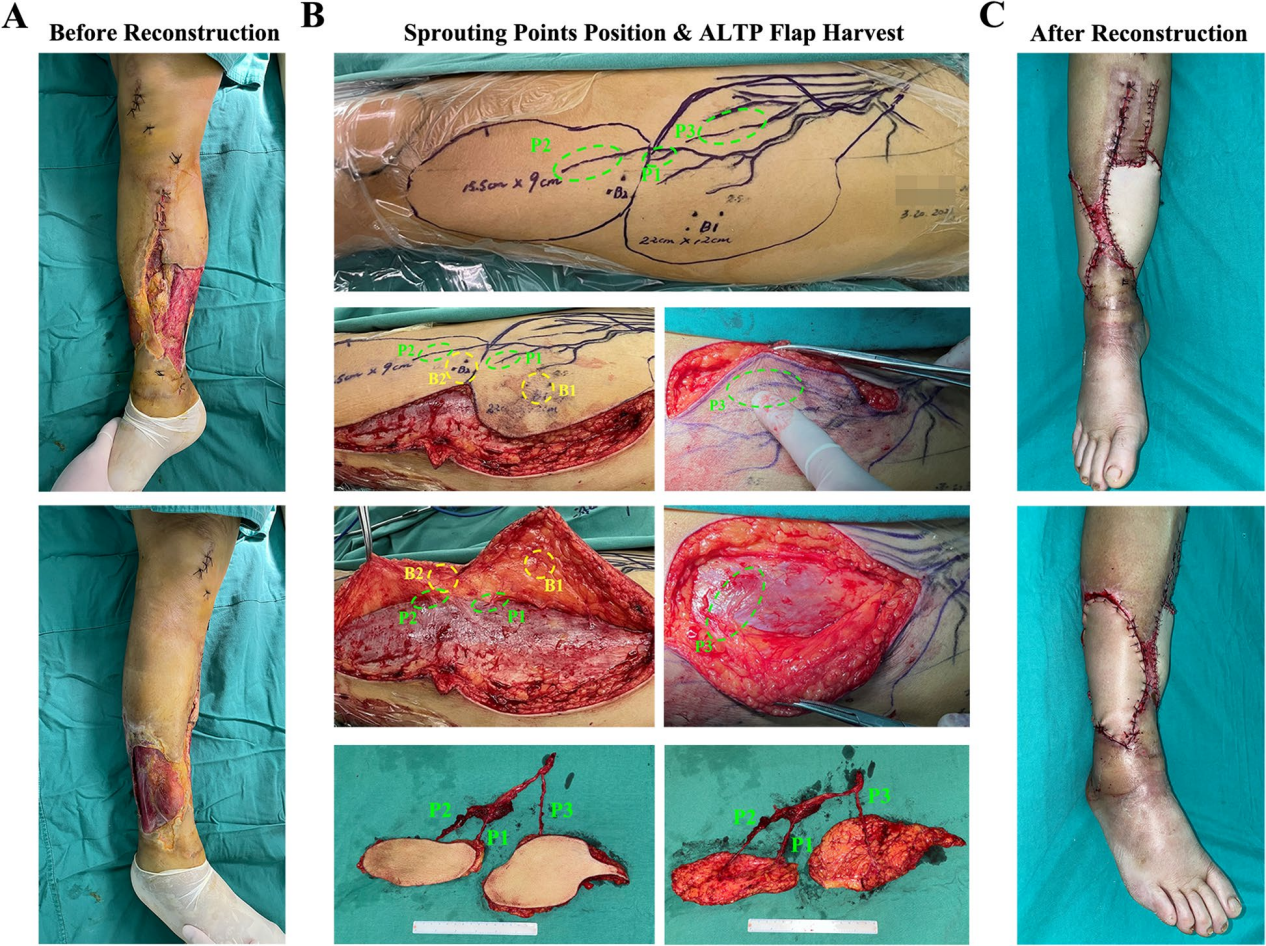
A typical case of a 52-year male. **A **After debridement, large soft tissue defects on the medial and lateral sides of the right calf existed, accompanied by tendon and bone exposure. **B **The location of the emerging point (P1-P3) came out from the deep fascia and the real-time perforators mapping were depicted on the anterolateral skin of the left thigh according to the prepared CTA images. Meantime, the pulse point of perforators (B1-B2) was also marked on the skin surface using handheld Doppler ultrasound. During operation, the lateral edge of the flap was cut up, and the cutaneous branches around the emerging points were carefully separated. It should be noticed that the emerging points of the perforators were in accordance with the preoperative marked locations determined by AR, while the points located by ultrasound were far away from the perforators. Finally, a double-foliate ALTP flap containing three perforators was obtained. **C** The damaged calf was reconstructed by the ALTP flap

**Fig. 7:**
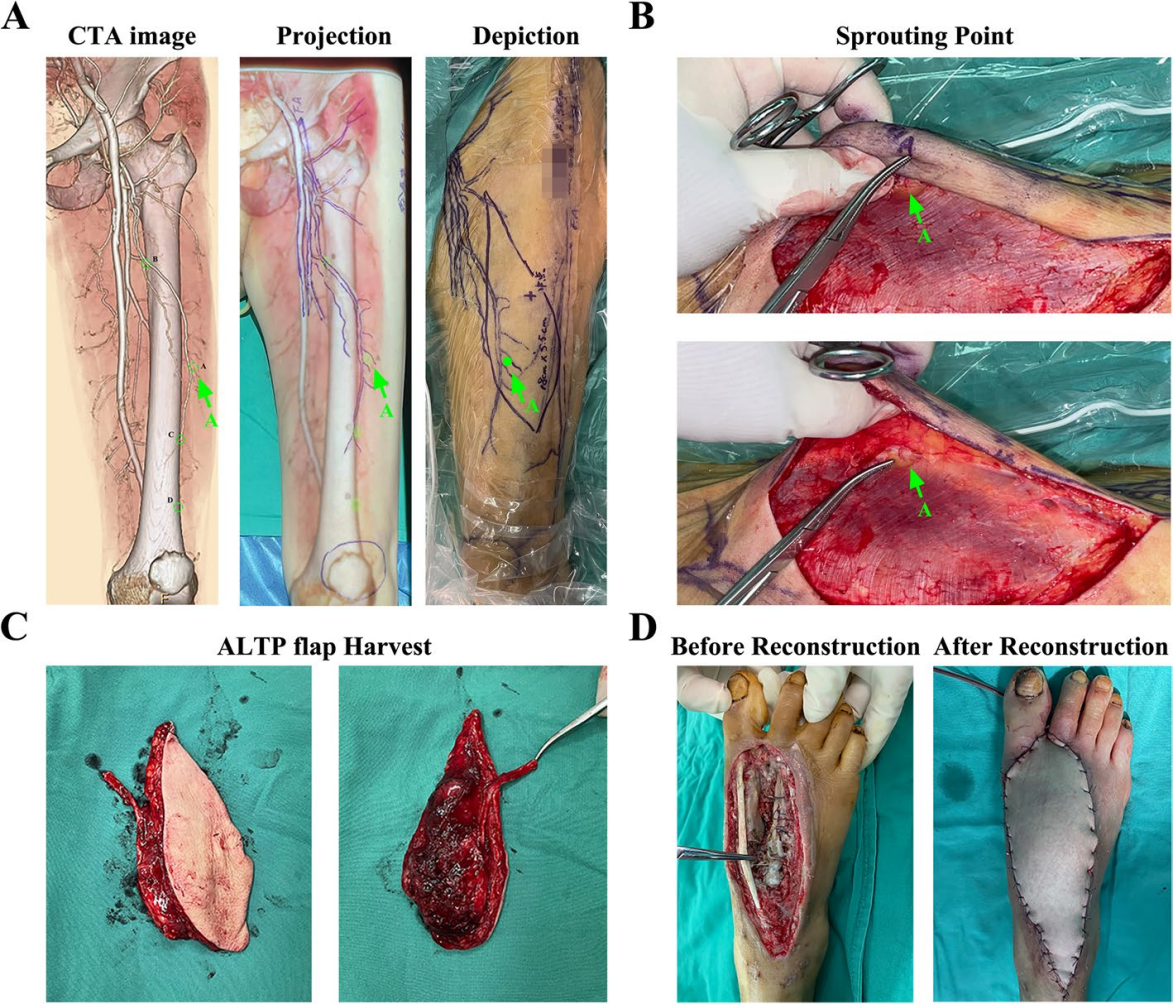
A typical case of a 56-year female. **A** Four marked emerging points A-D were showed in CTA images, but only one point was in the designed ALTP flap area. **B** During operation, the preoperatively marked point A was just above the emerging point of the perforator (green arrow). **C** The harvest ALTP flap and vascular pedicle. **D** After debridement, the damaged foot with 18.0 cm * 5.5 cm soft tissue defect was reconstructed by the ALTP flap

In the 14 patients, all ALTP flaps were successfully used to reconstruct soft tissue defects in the damaged limbs, with two patients suffering from complications, including partial flap necrosis and mixed infections (Table 1). In Figs. 6 and 7, two typical cases are displayed to illustrate the specific procedure and results of the CTA & AR projection technique applied in the clinic. Briefly, Fig. 6 shows the treatment procedure of patient 9. After thorough debridement, there were large soft tissue defects on both the medial and lateral sides of the right calf, with tendon and bone exposure. Moreover, the medial and lateral wounds were connected by a posterior subcutaneous tunnel. The wound was defined as Gustilo IIIB with a MESS of 6 points. After the preoperative CTA examination, a total of 3 emergence points of the ALTPs were marked, while 2 points were localized by Doppler ultrasound. Then, the flap was designed according to the preoperatively localized points, and the accuracy of these points was evaluated by intraoperative flap harvest. During the operation, a 15.5*9.0 cm and 22.0*12.0 cm double-leaf ALTP flap was obtained to repair the soft tissue defect in the right calf. Ultimately, the ALTP flap survived, and no related complications occurred. In another case, Fig. 7 shows the treatment procedure for patient 12. The right dorsal foot of this patient was unfortunately strangulated by a machine for chicken farming and was mixed with numerous chicken droppings, grains, and dirt. No improvements occurred after several debridements, and a soft tissue defect of approximately 18*5.5 cm was present on the dorsum of the right foot, with a dorsalis pedis artery defect and metatarsal exposure. Overall, the wound was defined as Gustilo IIIC with a MESS of 10 points. The bacterial culture showed a multidrug resistant mixed bacterial infection (Enterobacter cloacae & Proteus vulgaris). After thorough debridement, CTA was performed, and 4 emergence points (points A-D) of the ALTPs were marked, of which the thickest perforator was point A. Accordingly, the ALTP flap was designed and depicted on the skin surface, encompassing 3 ultrasound-located points. During the operation, a thick perforator emerging from the deep fascia under CTA-marked point A (indicated by the forceps) was seen to converge into the descending branch of the LCFA, whereas the ultrasound-marked perforators mostly continued into the transverse branch or traffic branches emanating from the deep femoral artery. Simultaneously, a portion of the rectus femoris and its corresponding musculocutaneous artery was obtained along with the flap to fill the foot defect, with the aim of controlling the severe infection. The flap eventually survived, but the wound continued to exude yellowish fluid after the operation, probably due to the bacterial infection. After being treated with anti-infection drugs and daily dressing changes, the wound healed completely after one month.

## Discussion

In this study, the ALTPs could be distinctly visible by optimizing scan parameters and prolonging the delay time during CT examination. Based on this, the muscle-subcutaneous fat interface, namely, the deep fascia, was identified and displayed by volume rendering. The emergence points, which indicated where the ALTPs went through the deep fascia and dispersed to subcutaneous tissue, were marked sequentially by checking every cross-section. This was the key point of this whole study. Then, the prelabeled CTA images were imported into a portable projector to depict the course of the ALTPs and the marked emergence points on the thigh surface of the donor area according to bony markers (ASIS and patella). Through verification with intraoperative findings, it was found that the CTA & AR projection technique was much more accurate and sensitive in detecting emergence points than handheld Doppler ultrasound. Moreover, the distances between the CTA-marked emergence points and the intraoperative findings were much smaller (*P* < 0.001), and the consistency was better (*P* = 0.037) than that in the handheld ultrasound group.

Among current perforator localization techniques, Doppler ultrasound is commonly used due to its low cost and high convenience [13]. Therefore, this technique was used for the comparison with the CTA & AR projection technique in this study, and many unavoidable drawbacks were exposed. First, the localization accuracy of ultrasound is extremely poor, and ultrasound cannot determine whether the vessel originates from the lateral circumflex femoral artery or other arteries. The localization accuracy is critically dependent on the proficiency and anatomical knowledge of the operator. In this study, the accuracy in the ultrasound group was 82.4% (28/34), which was lower than that in the CTA group. Moreover, the distance between the ultrasound-determined points and actual emergence points was 1.97 ± 0.50 cm, which was significantly further than that the 0.53 ± 0.27 cm in the CTA group (*P* < 0.001). In addition, handheld Doppler ultrasound could not determine the depth of the artery; thus, it was often confused by deep arterial pulsation, which is irrelevant, thus leading to bad judgments. In addition, the number of perforators was significantly lower than that detected by CTA, and in this study, the emergence points explored by ultrasound were only 69.1% (38/55) of those explored by CTA because many small perforators were easily overlooked. Furthermore, ultrasound was unable to accurately determine the course of the LCFA descending branch and its perforators. Therefore, it was not possible to specifically select the dominant perforators of the ALTP flap, while CTA could easily lead to the selection of a desired and thick perforator. Hence, the CTA examination has distinct benefits to assist in flap design and allows for improved flap survival.

To date, a remarkable number of studies have used CTA to display the courses, numbers, and locations of perforators [14, 15]. Despite the potential but acceptable radiation damage, the CTA image has a higher degree of accuracy and precision individually, which makes the perforator flaps harvest more efficiently. Among them, Hummelink used preoperative CTA and a handheld projector to project images of the deep inferior epigastric perforator (DIEP) onto the abdominal skin surface to obtain the DIEP flap [11]. In parallel, Shen used CTA to display the whole course of the LCFA and its branches by volume rendering. Then, a 1:1 template was printed out and overlapped on the donor site to mark the perforator points and finally obtain an ALTP flap [10]. Both methods can not only allow for more perforators to be identified intraoperatively but also save time during flap harvesting. However, flap surgery is labor intensive and time-consuming for many microsurgeons, making it difficult to maintain peak performance and leading to iatrogenic perforator injury and errors during flap harvesting. As the surgeon often segments the ALTP flap from the muscle or deep fascia layer, the perforators emerging from the deep fascia may be the most frequent injury site [2]. Thus, localization of the emergence point is crucial. On the other hand, according to the anatomical characteristics of the subcutaneous vascular network, the flap is centered on the emergence point, and the perforators penetrate to the superficial subcutaneous layer vertically and radiate out to the flap edge [16]. Therefore, the AR technique applied in this study plays a vital role in marking the emergence points accurately and facilitating flap harvest, meaning that the flap design no longer needs to be arbitrary or empirical.

Currently, there are also many novel methods to localize perforators. Debelmas believes that preoperative color Doppler ultrasound is reliable, accurate, and compatible with a quick routine assessment [17]. However, this method is more dependent on the proficiency of the operator, and it does not have the advantage of providing a holistic view of the source, route, and feature of each perforator. Moreover, Pereira successfully applied a thermographic imaging technique to detect the distribution of perforators in the anterolateral thigh, despite its poor accuracy [18]. Unfortunately, it is easy for this method to overlook smaller perforators and miss deep perforator information, making it a complementary tool to explore perforators. The HoloLens is a more accurate and advanced device that can partially eliminate the subtle errors caused by space variation. This device is expected to become an important tool to aid surgeries in the future. However, the high cost of the equipment and its relatively tedious operation limit its application and promotion [19]. As a specialty that requires surgeons to frequently extrapolate 3D data from 2D imaging, AR technology is likely to have multiple applications in reconstructive microsurgery. The purpose of this technology is to minimize distractions and help surgeons maintain reference points by placing imaging data and other relevant information within the surgical area [20]. In several recent studies, intraoperative perforator identification in plastic surgical procedures was found to be more accurate with AR than with other methods [5, 21–23]. In general, CTA & AR projection technology could be an accurate and convenient tool to aid in harvesting ALTP flaps at present.

Despite numerous advantages, a few limitations remain: 1. Some errors might exist in the overall procedure, as small tissue shifts can create errors spanning a few mm/cm errors. Muscles must be relaxed during the operation, but they maintain normal tension during the CT scan. This creates tissue positional discrepancy. 2. During the projection process, the 2D image will be reflected on the curved thigh surface so the corresponding marked points will be slightly shifted. It is expected that the curved surface will be automatically scanned and processed by the corresponding algorithm before projection to improve its accuracy. 3. Critics of CTA mapping have raised questions regarding the acquisition of high-resolution CT data and cost. Although CTA is more popular than before and has become affordable at a lower price, it is still significantly more expensive than portable ultrasound. 4. CTA is an invasive examination that requires a higher contrast agent volume with a rapid infusion rate to show tiny vessels. Although the dose is still safe, it may have potential adverse effects on patients suffering from renal insufficiency. In this study, 14 cases were involved, and the success rate of locating perforators using ultrasound may vary from the rate in other studies due to the ultrasound proficiency of the operators. However, it can be affirmed that the sensitivity and accuracy of mapping the perforator emergence points with the CTA & AR projection method are extremely high, indicating that this approach is more dependent on scanning parameters and requires less imaging expertise, which makes it easy to promote.

## Conclusion

The localization technique for the perforator emergence points is of vital importance in flap harvesting but has rarely been explored. In this study, we used augmented reality, namely, CTA combined with a portable projection technique, to depict the course of the ALTPs and their emergence points on the anterolateral thigh, achieving a significantly higher accuracy and sensitivity than portable ultrasound. Potentially, this method may facilitate the individualized preoperative design of multiple forms of flaps and ensure that the dominant perforators are preserved and distributed in the desired location. Furthermore, this technique can guarantee the blood supply of the flap, avoid unnecessary soft tissue stripping, and finally promote flap survival. In the future of microsurgery, it can be expected that many promising developments will arise, such as automatic bony marker localization, holographic projection, or even fully automated flap harvesting performed by robots after importing CTA information.

## Data Availability

All relevant raw data supporting the conclusions were included within the article and tables.
